# Ontogeny of Recognition Specificity and Functionality for the Broadly Neutralizing Anti-HIV Antibody 4E10

**DOI:** 10.1371/journal.ppat.1004403

**Published:** 2014-09-25

**Authors:** Kathryn A. K. Finton, Della Friend, James Jaffe, Mesfin Gewe, Margaret A. Holmes, H. Benjamin Larman, Andrew Stuart, Kevin Larimore, Philip D. Greenberg, Stephen J. Elledge, Leonidas Stamatatos, Roland K. Strong

**Affiliations:** 1 Division of Basic Sciences, Fred Hutchinson Cancer Research Center, Seattle, Washington, United States of America; 2 Department of Genetics, Harvard University Medical School, and Division of Genetics, Howard Hughes Medical Institute, Brigham and Women's Hospital, Boston, Massachusetts, United States of America; 3 Seattle Biomedical Research Institute, Seattle, Washington, United States of America; 4 Department of Immunology, University of Washington, Seattle, Washington, United States of America; 5 Department of Medicine, University of Washington, Seattle, Washington, United States of America; 6 Program in Immunology, Cancer Research Division, Fred Hutchinson Cancer Research Center, Seattle, Washington, United States of America; 7 Department of Global Health, University of Washington, Seattle, Washington, United States of America; Duke University Medical Center, United States of America

## Abstract

The process of antibody ontogeny typically improves affinity, on-rate, and thermostability, narrows polyspecificity, and rigidifies the combining site to the conformer optimal for binding from the broader ensemble accessible to the precursor. However, many broadly-neutralizing anti-HIV antibodies incorporate unusual structural elements and recognition specificities or properties that often lead to autoreactivity. The ontogeny of 4E10, an autoreactive antibody with unexpected combining site flexibility, was delineated through structural and biophysical comparisons of the mature antibody with multiple potential precursors. 4E10 gained affinity primarily by off-rate enhancement through a small number of mutations to a highly conserved recognition surface. Controverting the conventional paradigm, the combining site gained flexibility and autoreactivity during ontogeny, while losing thermostability, though polyspecificity was unaffected. Details of the recognition mechanism, including inferred global effects due to 4E10 binding, suggest that neutralization by 4E10 may involve mechanisms beyond simply binding, also requiring the ability of the antibody to induce conformational changes distant from its binding site. 4E10 is, therefore, unlikely to be re-elicited by conventional vaccination strategies.

## Author Summary

4E10 is an antibody that neutralizes a broad variety of HIV strains. However, 4E10 is uncommon in infected patients and has not been successfully elicited by any vaccine approach attempted. Hurdles to re-eliciting 4E10 include the accumulation of many mutations during development, demonstrated reactivity against host proteins and significant structural flexibility. Lacking a confirmed sequence for precursors of 4E10, we studied the recognition and biophysical properties of an ensemble of eight of the likeliest candidates. Surprisingly, 4E10 gained host reactivity and structural flexibility, but lost stability during development when compared to candidate precursors. However, recognition of HIV was remarkably conserved, despite a considerable improvement in binding. Since these results run counter to those expected from conventional vaccination protocols, 4E10 is unlikely to serve as the basis of a useful HIV vaccine.

## Introduction

An effective HIV vaccine will likely need to elicit broadly-neutralizing antibodies (bnAbs) that target the viral envelope protein (Env) as part of a protective immune response [1]–[6]. Env-derived and reverse-engineered immunogen-based vaccines, however, have consistently failed to elicit bnAbs. Possible explanations include that: (1) immunogens may be unable to bind germline-encoded precursors (GEPs) of bnAbs with sufficient affinity to initiate B cell activation and affinity maturation, which has a ∼micromolar threshold [7]–[9]; (2) rearranged V_H_ and V_L_ genes compatible with the development of bnAbs may not be common in human or animal model vaccinee GEP repertoires; (3) some bnAbs are autoreactive, which hinders their elicitation through self-tolerance mechanisms; (4) the unusual characteristics inherent to bnAbs, such as long complementarity determining regions (CDRs), functionally-required polyspecificity, and a high degree of somatic mutation (typically observed in Abs elicited in response to chronic infections, including bnAbs), may not be easily achieved through conventional vaccination strategies; (5) imperfect immunogens may elicit off-target (non-neutralizing or non-Env) or humoral responses with limited breadth; and, finally, (6) neutralization mechanisms may involve complexities beyond simply binding a particular epitope on Env (e.g., inducing specific conformational changes), which may be difficult to recapitulate, since selective expansion of particular B cell clones is based solely on BCR binding properties, not higher-order functionalities [10], [11].

The bnAb 4E10, the focus of our studies, has a conserved, linear epitope (core epitope: ^671^NWF^D^/_N_IT^676^) immediately adjacent to the viral membrane in the Env gp41 subunit membrane proximal external region (MPER) [12], [13]. While 4E10 displays admirable breadth [14], has been the target of a successful design effort to reverse-engineer tight-binding immunogens [15], has recognizable GEPs present at finite frequencies in human naïve repertoires [16], and arguably does not require a high degree of polyspecificity to neutralize HIV [17], [18], its viability as a vaccine target is hampered by limited potency, demonstrated autoreactivity and exceptional combining site flexibility [17], [19], [20]. The neutralization mechanism of 4E10 also has not been clearly defined and may involve higher order effects [17], [21], [22]. The ontogeny of 4E10, therefore, must be elucidated in order to understand how these properties were acquired and to what degree they impose constraints that might hinder re-elicitation by vaccination.

**Figure 1:**
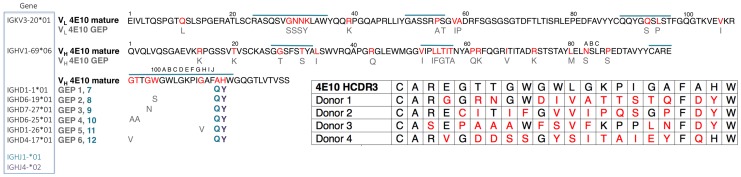
Prediction of an ensemble of 4E10 GEPs. Sequences of 4E10 V_L_ (*top line*) and V_H_ (*bottom two lines*) domains are shown, with CDRs indicated by a blue overscore. Predicted somatic mutations are colored red in the 4E10 sequence, and the corresponding unmutated GEP residues are shown below in grey (unchanged positions are not shown for clarity). All GEP V_L_ domains are comprised of the IGKV3-20*01/IGKJ1*01 gene segment combination. Each GEP V_H_ domain comprises the IGHV1-69*06 V gene segment plus one of six D gene segments (listed to the left of the corresponding GEP in the blue boxed field), and either the IGHJ4*02 (resulting sequence differences shown in purple and bolded) or IGHJ1*01 (resulting sequence difference shown in blue and bolded) gene segment, yielding an ensemble of 12 GEPs *in toto*. GEP shorthand numbering is shown in grey (GEPs 1 to 6) and blue (GEPs 7 to 12) beside the corresponding D plus J gene segment combination sequence differences from 4E10, occurring in HCDR3. *Inset* (*lower right*): HCDR3 sequences from candidate 4E10 GEPs, determined through deep sequencing of naïve B cell germline IgH genes from four uninfected individuals [16], show the degree of variability seen in potential 4E10 precursors present in naïve repertoires. Each germline rearrangement uses the IGHV1-69 and IGJH1 or IGJH4 gene segments. Amino acids in red designate sequence differences between GEP and 4E10. The number of nucleotide changes needed to achieve these somatic mutations is 16 for donor 1, 17 for donor 2 and 18 for donors 3 and 4.

Mutations acquired during Ab maturation occur preferentially in the CDRs, which make up the six loops (CDRs 1, 2, and 3 on the heavy (HCDRs) and light (LCDRs) chains) comprising the combining site [23], [24]. CDRs are responsible for the majority of direct contacts to an antigen, as opposed to the intervening framework regions (FWRs), which form the immunoglobulin β-sheet structure stabilizing the combining site, helping define CDR loop conformations. While CDR mutations are typically thought to more directly affect antigen binding and neutralization, bnAbs consistently depend on FWR substitutions to a surprising degree, though 4E10 is an exception to this exception [4]. bnAbs are notorious for their high degree of somatic hypermutation, the product of a long process of affinity maturation against a rapidly mutating virus during a persistent, chronic infection [25]. While typical affinity-matured Abs have acquired 15 to 20 V_H_ mutations, bnAbs accumulate up to 100 V_H_ mutations [4]. These mutations are crucial because reversion to germline sequences drastically reduces epitope affinity and neutralization potency and breadth. In many cases, bnAb GEPs are unable to bind Env, though the actual eliciting isolate may not be known [26]–[31]. In addition, bnAbs can contain extraordinarily long HCDR3s, up to 33 residues long versus an average of 13 for non-HIV bnAbs [32], [33]. Using phylogenetically-inferred GEP sequences [16], [34] (**Fig. 1**), 4E10 has acquired between 33 and 35 mutations during maturation, 20 to 22 in V_H_, depending on gene segment selection, and 13 in V_L_, and has an HCDR3 22 residues long, values at the less exceptional end of the bnAb spectrum and not unheard of for conventional Abs.

Affinity-matured Abs display univalent equilibrium binding constants (*K*_D_) for their cognate antigens, typically ranging from 10^−6^ to 10^−10^ M, that are orders-of-magnitude stronger than their GEPs [35]. Multiple approaches, including computational analyses and biophysical comparisons of affinity-matured Abs and their associated GEPs, have generated a consensus model for the molecular mechanism of affinity maturation [36]–[47], perhaps better understood as binding optimization, that traces its roots back to Pauling [48]. In the consensus model, the antigen specificities of the naïve, germline-encoded repertoire, while diverse and extensive, are further extended by encoding a high degree of polyspecificity into GEPs. This is accomplished partly through increased combining site plasticity in GEPs, more formally stated as the ability of GEP CDRs to dynamically sample a broader ensemble of structural conformers. In response to immunogen stimulation, Ab binding properties are iteratively optimized through cycles of somatic hypermutation and selection, resulting in improved binding affinities, kinetics and thermodynamics. While mutations have been observed to improve or add direct contacts to antigen, typically improving enthalpies of interaction and off-rates (*k*_d_), a majority of measurably favorable mutations do not directly contact antigen. These mutations indirectly optimize binding by: (1) increasing shape complementarity between paratopes and epitopes through more global effects on structure; (2) increasing antibody stability, typically measured as solution thermostabilities (T_m_), thus compensating for deleterious effects of other mutations that improve affinity; and (3) structurally rigidifying the combining site conformer optimal for binding antigen from the accessible ensemble. Rigidifying the combining site can affect measured interaction parameters in different ways depending on the mechanism of binding. The two mechanistic extremes are known as “conformer-selection” and “instructive-encounter”, or “induced-fit”, binding. In conformer-selection mode, binding does not occur until the compatible conformer is adopted. Rigidification of the binding site through mutation then typically improves entropies of interaction and on-rates (*k*_a_). In instructive-encounter mode, initial binding occurs to sub-optimal conformers which affects the rate of interchange with the optimal conformer. Rigidification of the binding site through mutation then typically improves affinity through changes distributed over *k*_d_ and *k*_a_. However, the consensus of studies of binding proteins and enzymes suggests that conformer selection is the preeminent recognition mechanism [49].

Surprisingly, comparisons of the bound and unbound structures of 4E10 revealed that this affinity-matured bnAb incorporates considerable HCDR conformational flexibility [17], in excess of what has been observed in most other antibodies, mature or GEP, suggesting that the ontogeny of 4E10 may be an exception to the consensus model and may pose unique challenges as a vaccine target. In order to fully understand the ontogeny of this unique bnAb and consequences for vaccine development, we characterized the unbound and complex crystal structures, and the functional and binding properties, of 4E10 and an ensemble of its most likely GEPs. GEPs showed detectable, but extremely weak, binding to soluble Env gp140s and extremely limited neutralization potency, though some reverse engineered epitope-scaffolds (ESs) showed robust GEP affinities, well above the B cell activation threshold. 4E10 and GEP paratopes displayed a remarkable degree of structural conservation in the antigen-bound state, with little improvement in overall shape complementarity. Multi-log improvements in affinity for ESs were the result of improved off-rates or combined improvements in on- and off-rates, with a small number of enhanced contacts to antigen observed in the crystal structures. However, minimal mutations of GEP sequences to include these enhanced direct contacts only marginally increases affinity. FWR mutations had little discernable effect on global or local structure. Controverting the consensus model of ontogeny, 4E10 thermostability was appreciably worse than its GEPs; while 4E10 and GEPs displayed similarly constrained V_H_/V_L_ interdomain movements upon binding, 4E10 maturation involved negligible combining site rigidification, with both 4E10 and GEP HCDRs sampling extensive conformer ensembles. The narrowing of polyspecificity assumed to concur with maturation was not observed with 4E10, as both 4E10 and its GEPs showed similar patterns of limited polyspecificity to a phage-displayed human peptidome (Phage Immunoprecipitation Sequencing; PhIP-Seq) [50]. While 4E10 is demonstrably autoreactive, GEPs exhibited a distinct profile of autoantigen recognition by PhIP-Seq. When combined, these results inform efforts to re-elicit 4E10 by vaccination and its mechanism of neutralization.

## Results

### Prediction of an ensemble of likeliest GEP sequences

Lacking access to the original donor, identification of a single GEP sequence with high confidence for many bnAbs, including 4E10, is complicated by extensive editing and TdT N-nucleotide insertion during rearrangement [26], leading to our decision to study an ensemble of the 12 likeliest candidates (**Fig. 1**). Due to the prediction that the sequence differences introduced by alternate heavy chain J gene segments may not affect any discernable GEP property, the initial ensemble was limited to eight GEPs (IGHJ4*02 paired with all six D segments plus IGHJ1*01 paired with IGHD1-1*01 and IGHD6-19*01), with the intention of generating additional GEPs if the IGHJ4*02/IGHJ1*01 substitution exhibited any differences in structure or binding properties on the IGHD1-1*01 or IGHD6-19*01 backgrounds. These eight GEPs also recapitulated some of the variability seen in potential 4E10 GEPs identified by deep sequencing of uninfected individuals (**Fig. 1**) [16], providing an additional justification for studying the ensemble. However, GEPs in our ensemble differed from one another by no more than four mutations, though the mutations were quite non-conservative.

### GEP protein production and validation

**Figure 2:**
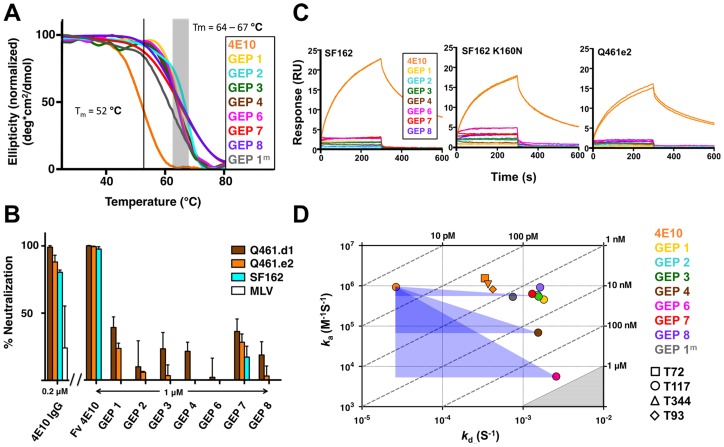
Biophysical and functional characterization of an ensemble of 4E10 GEPs. (**A**) CD melting curves are shown for 4E10 and GEPs with T_m_ values, determined as the inflection point of the sigmoidal melting curve, indicated by a black line (4E10) or a shaded grey box (GEPs). (**B**) Neutralization potencies are shown for 4E10 IgG (0.2 µM), 4E10 Fv and GEPs (1 µM) against clade A (Q461.d1, Q461.e2) and B (SF162) HIV-1 isolates using standard TZM-bl assays. (**C**) Double-referenced SPR sensorgrams are shown for the binding of 4E10 and GEPs (300 nM duplicates) interacting with chip-coupled gp140_3_. (**D**) *K*_D_s for the interaction of 4E10 or GEPs with ES T117 are plotted as *k*_a_ vs. *k*_d_, with *K*_D_ isotherms indicated by dashed lines and labeled. Due to weak binding and fast kinetics, *K*_D_s between GEPs and the T72, T344, and T93 ESs could only be analyzed by equilibrium measurements; their values range from 1 to 10 µM, falling within the grey shaded region. The purple triangles show the T117 affinity shift between 4E10 and GEPs, with the sides parallel to the X and Y axes of each triangle highlighting the association and dissociation rate components, respectively.

GEPs were engineered as cleavable, single-chain, variable domain cassette (Fv; V_H_+V_L_) constructs to ease expression, analysis, and crystallization and to prevent monobody-diabody interchange, following protocols developed for 4E10 [18]. The prior study confirmed that these Fv constructs retained the structural and binding properties of Fab fragments of intact 4E10. All eight GEP Fvs expressed at high levels as bacterial inclusion bodies and, in all but one case (GEP 5), were refolded *in vitro* with what in our experience were exceptionally high efficiencies of 20 to 40%. GEP 5 was not included in subsequent experiments because its extremely poor *in vitro* refolding efficiency suggested that this was not a viable pairing. GEP constructs were stable and monodisperse in solution, running exclusively as monomers by size exclusion chromatography (SEC). Reduced/non-reduced PAGE analysis of GEPs confirmed purity and proper disulfide bond formation. GEP T_m_ values (**Fig. 2A**), determined by circular dichroism (CD) spectroscopy as previously described [18], narrowly ranged from 64.2°C to 67.0°C, showing that GEP Fvs were well folded, but had even higher T_m_ values than 4E10 Fv (52.8°C).

### GEP neutralization potencies were dramatically reduced

The neutralization potency of GEPs was tested against clade A (Q461.d1, Q461.e2 [51]) and B (SF162 [52]) HIV-1 isolates using standard TZM-bl assays (**Fig. 2B**) [53]. Overall, GEP Fv potencies were markedly reduced in comparison with 4E10 Fv. GEPs displayed only very weak and likely insignificant neutralization potencies, though with a trend of greater effect against the clade A isolates, particularly the neutralization-sensitive strain Q461.d1, and with GEP 1 and GEP 7 showing marginally better potencies across tested isolates.

### GEPs bound Env proteins and engineered antigens, but with reduced affinities

**Figure 3:**
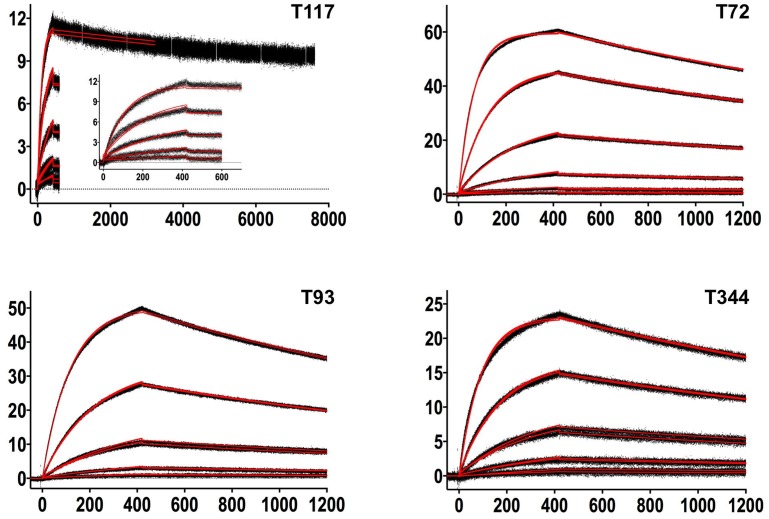
SPR sensorgrams of the interactions between 4E10 and the indicated ESs are shown. Time (in seconds) is plotted on the x-axis and SPR response (in RUs) is plotted on the y-axis. Double-referenced binding data are shown in black with corresponding kinetics fits to the data shown in red. Details of the experiments are given in Table 1.

**Figure 4:**
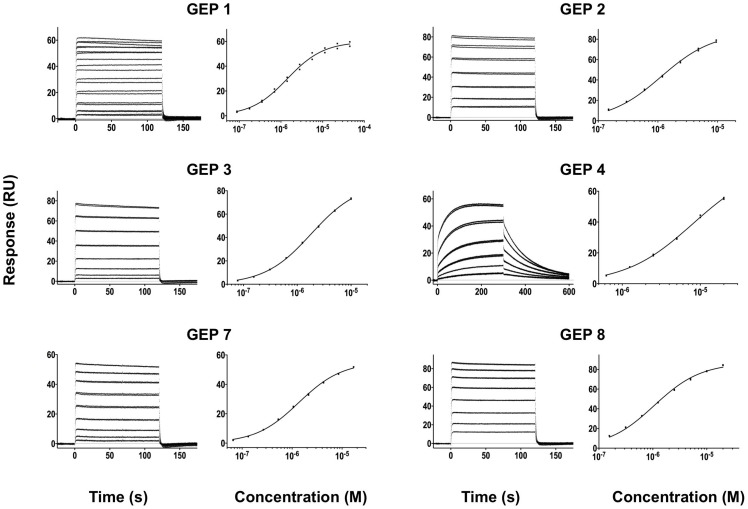
SPR sensorgrams of the interactions between ES T72 and the indicated GEPs (left plots) and the analysis of equilibrium responses versus concentration (right plots). Details of the experiments are given in Table 1.

**Figure 5:**
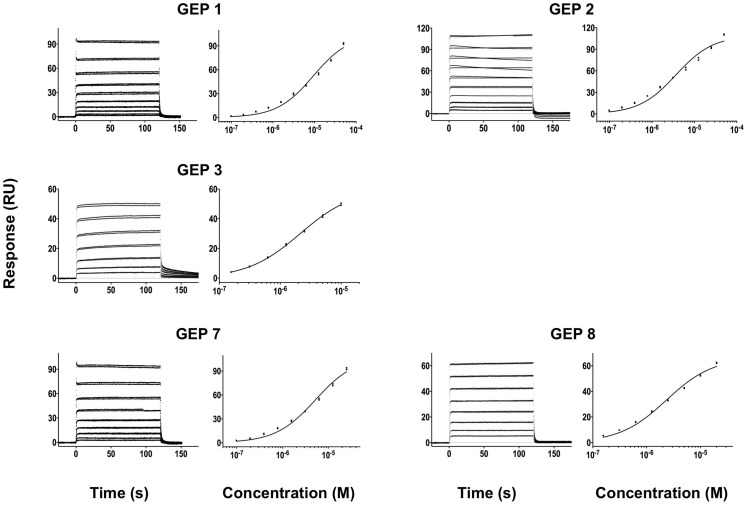
SPR sensorgrams of the interactions between ES T93 and the indicated GEPs (left plots) and the analysis of equilibrium responses versus concentration (right plots). Details of the experiments are given in Table 1.

**Figure 6:**
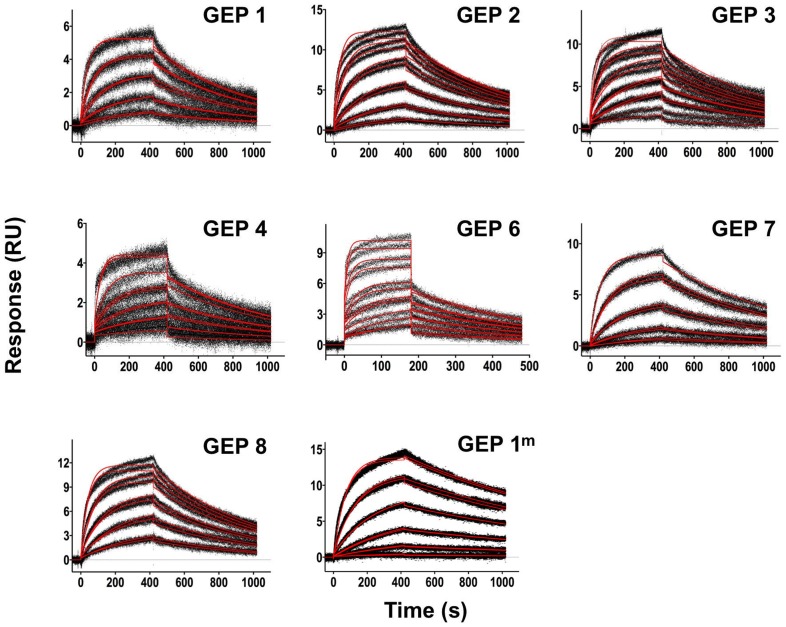
SPR sensorgrams of the interactions between ES T117 and the indicated GEPs are shown. Binding data are shown in black with the kinetic fits to the data shown in red. Details of the experiments are given in Table 1.

**Figure 7:**
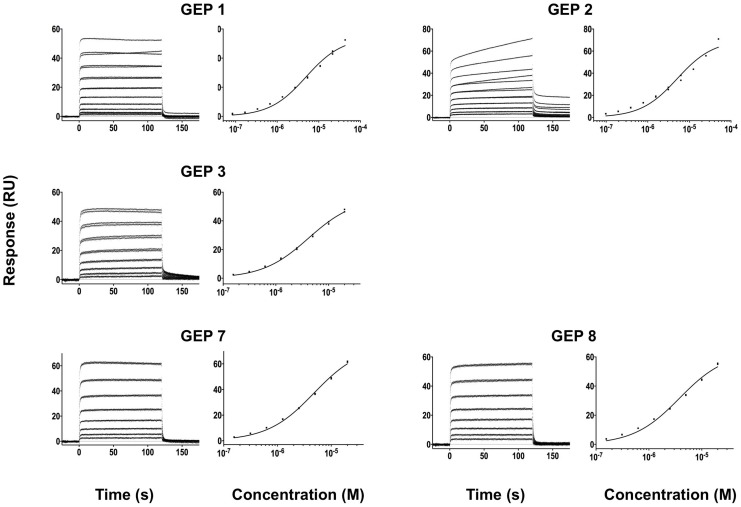
SPR sensorgrams of the interactions between ES T344 and the indicated GEPs (left plots) and the analysis of equilibrium responses versus concentration (right plots). Details of the experiments are given in Table 1.

In order to characterize the change in binding properties during 4E10 ontogeny, the binding of 4E10 and GEP Fvs to three (SF162, SF162^K160N^ and Q461.e2) soluble Env gp140 trimers (gp140_3_) [54] and four engineered 4E10 ES immunogens (T72, T93, T117 and T344) [15], [55] was evaluated by surface plasmon resonance (SPR) interaction analysis (**Figs. 2C, 2D**, **Figs. 3**–**7**, **Table 1**). Isolates and ESs were selected to span a range of binding properties, where previous studies had shown 4E10 bound a free peptide spanning its linear epitope with a *K*_D_ of 12 nM, SF162 gp140_3_ with a *K*_D_ of 98 nM, and ESs with *K*_D_s of either ≤10 picomolar (T117) or ∼100 picomolar (T72, T93 and T344) [15],[18],[55]. All seven GEPs showed unquantifiably weak, but detectable binding to chip-coupled clade A (Q461.e2) and clade B (SF162, SF162^K160N^) gp140_3_ and T72, T93, and T344 in qualitative SPR analyses, with *K*_D_s all estimated to be well above the ∼micromolar B cell activation threshold. Quantitative SPR analyses of GEPs binding to T117 showed *K*_D_s ranging from the low nanomolar to low micromolar range. 4E10 interactions with ESs ranged from 100- to 10,000-fold stronger than GEPs, which was qualitatively consistent with the observed difference in 4E10 versus GEP interactions with gp140_3_. Since the GEP/gp140_3_ interactions were too weak to quantitate, peptide binding studies were not performed on the expectation that they would also be too weak to measure accurately. Five GEPs (1, 2, 3, 7 and 8) bind T117 with nearly identical behavior, including GEP 1 and GEP 7, which differ only by alternate J segment utilization, showing that the two incorporated mutations did not affect binding, so no further IGHJ1*01 GEPs were produced for study. GEP 4 and GEP 6, which both incorporate differences from G96H in 4E10 (A or V), showed approximately 10-fold (GEP 4) or 100-fold (GEP 6) reductions in affinity relative to the cluster of other GEPs. Kinetically, the five clustering GEPs (1, 2, 3, 7 and 8) showed affinity reductions for T117 relative to 4E10 overwhelmingly through faster off-rates (*k*_d_s). GEPs 4 and 6, in addition to comparable increases in *k*_d_, also showed progressive reductions in on-rates (*k*_a_s).

**Table 1:** SPR methods and results.

Ligand		Analyte					Results		
	(RU)		Concentration Range	Injection Time (s)	Dissociation Time (s)	Regeneration	*k*_a_ (M^−1^s^−1^)	*k*_d_ (s^−1^)	*K*_D_ (M)
**T117**	129	**4E10**	10 - 0.625 nM	420	180 or 7200	2×10 s, pH 1.5	9.35(2)×10^5^	2.65(1)×10^−5^	2.83(1)×10^−11^
**T117**	129	**GEP 1**	50 - 3.12 nM	420	600	5 s, pH 1.5	4.56(2)×10^5^	1.80(1)×10^−3^	3.96(2)×10^−9^
**T117**	183	**GEP 2**	50 - 1.56 nM	420	600	5 s, pH 2.0	5.90(1)×10^5^	1.52(1)×10^−3^	2.57(1)×10^−9^
**T117**	86	**GEP 3**	50 - 0.78 nM	420	600	5 s, pH 1.75	5.43(1)×10^5^	1.56(1)×10^−3^	2.88(1)×10^−9^
**T117**	129	**GEP 4**	400 - 12.5 nM	420	600	5 s, pH 1.5	6.90(4)×10^4^	1.54(1)×10^−3^	2.23(1)×10^−8^
**T117**	129	**GEP 6**	20 - 0.625 µM	180	300	none	5.61(3)×10^3^	2.58(1)×10^−3^	4.60(3)×10^−7^
**T117**	183	**GEP 7**	25 - 1.56 nM	420	600	5 s, pH 2.0	6.31(2)×10^5^	1.30(1)×10^−3^	2.06(1)×10^−9^
**T117**	86	**GEP 8**	25 - 1.56 nM	420	600	5 s, pH 1.75	9.18(2)×10^5^	1.63(1)×10^−3^	1.78(1)×10^−9^
**T117**	122	**GEP 1 m**	25 - 0.78 nM	420	600	2×10 s, pH 1.5	5.33(1)×10^5^	7.47(1)×10^−4^	1.40(1)×10^−9^
**T72**	30	**4E10**	10 - 0.312 nM	420	800	10 s, pH 1.5	1.54(1)×10^6^	3.36(1)×10^−4^	2.19(1)×10^−10^
**T72**	30	**GEP 1**	45 - 0.088 µM	120	180	none			1.4(1)×10^−6^
**T72**	53	**GEP 2**	9.5 - 0.149 µM	120	180	none			1.25(6)×10^−6^
**T72**	53	**GEP 3**	10 - 0.078 µM	120	180	none			1.78(4)×10^−6^
**T72**	53	**GEP 4**	20 - 0.625 µM	300	300	10 s, pH 1.5			7.9(5)×10^−6^
**T72**	30	**GEP 6**	1 µM	120	180	none			*None detected*
**T72**	30	**GEP 7**	17 - 0.066 µM	120	180	none			1.34(5)×10^−6^
**T72**	53	**GEP 8**	20 - 0.156 µM	120	180	none			1.26(6)×10^−6^
**T93**	49	**4E10**	10 - 0.625 nM	420	800	10 s, pH 1.5	8.22(1)×10^5^	4.21(1)×10^−4^	5.13(1)×10^−10^
**T93**	75	**GEP 1**	50 - 0.098 µM	120	180	none			9.3(1)×10^−6^
**T93**	49	**GEP 2**	50 - 0.098 µM	120	180	none			5.1(7)×10^−6^
**T93**	27	**GEP 3**	10 - 0.156 µM	120	180	none			2.2(1)×10^−6^
**T93**	27	**GEP 4**	20 - 0.625 µM	300	300	10 s, pH 1.5			*None detected*
**T93**	75	**GEP 6**	1 µM	120	180	none			*None detected*
**T93**	75	**GEP 7**	25 - 0.098 µM	120	180	none			5.23(6)×10^−6^
**T93**	27	**GEP 8**	20 - 0.156 µM	120	180	none			3.1(3)×10^−6^
**T344**	28	**4E10**	10 - 0.625 nM	420	800	10 s, pH 1.5	1.08(1)×10^6^	3.67(1)×10^−4^	3.41(1)×10^−10^
**T344**	28	**GEP 1**	43 - 0.084 µM	120	180	none			6.4(6)×10^−6^
**T344**	28	**GEP 2**	50 - 0.098 µM	120	180	none			8(1)×10^−6^
**T344**	35	**GEP 3**	20 - 0.156 µM	120	180	none			4.8(3)×10^−6^
**T344**	35	**GEP 4**	20 - 0.625 µM	300	300	10 s, pH 1.5			*None detected*
**T344**	35	**GEP 7**	20 - 0.156 µM	120	180	none			5.7(4)×10^−6^
**T344**	35	**GEP 8**	20 - 0.156 µM	120	180	none			5.2(4)×10^−6^

### 4E10- and GEP-ES complex structures show binding site conservation

**Figure 8:**
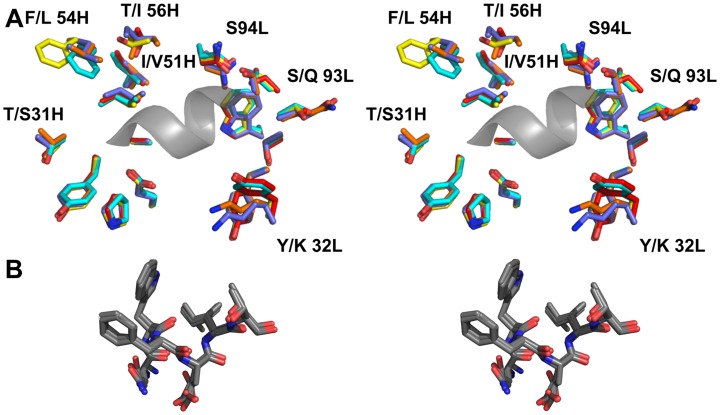
The epitope binding site is conserved between 4E10 and its GEPs. (**A**) Residues from the combining sites of 4E10 and GEPs, superimposed based on bound-state structures, contacting the NWFDIT core epitope (shown in a cartoon representation as a grey corkscrew) are shown in a stereo view. 4E10 and GEP residues are shown in licorice stick representation and colored as follows: 4E10 Fv (3LH2.pdb, in complex with ES T88) in purple, 4E10 Fab (2FX7.pdb, in complex with an extended 16-mer NWFDIT-containing peptide) in orange, GEP 1 in yellow, GEP 2 in cyan, and GEP 7 in red. Analyses and depictions were restricted to the core epitope to maximize comparability. Calculated SA buried in the complexes and Sc between core epitope and Ab are: 4E10 Fab/epitope peptide: SA = 355 Å^2^, Sc = 0.78; 4E10/T88: SA = 367 Å^2^, Sc = 0.77; GEP 1/T117: SA = 342 Å^2^, Sc = 0.69; GEP 2/T117: SA = 305 Å^2^, Sc = 0.74; GEP 7/T117: SA = 343 Å^2^, Sc = 0.66. (**B**) The NWFDIT core epitope is shown in a licorice stick representation isolated from the superimposed complex structures, highlighting the high degree of conservation of both the position and conformation of the peptide across 4E10 and GEP complexes.

In order to shed light on potential structural differences accounting for reduced GEP binding affinities, crystal structures of GEP 1, 2 and 7 in complex with T117 were determined at resolution values of 2.9 Å, 1.8 Å, and 3.1 Å respectively, rebuilt and refined with good statistics (**Table 2**), and compared to two reference 4E10/antigen complex structures: 4E10 bound to an epitope peptide (2FX7.pdb [12]) or a related ES, T88 (3LH2.pdb [15]). Superpositions showed that almost all direct contacts to the core epitope (NWFDIT) and epitope conformation are conserved between 4E10 and GEP complexes to a remarkable degree (**Fig. 8**, **Tables 3** and **4**). Only six of the 35 predicted somatic mutations affect sequence positions making direct contacts to the core epitope: Y/K32L, S/Q93L, T/S31H, I/V51H, F/L54H and T/I56H. However, residues at two of these positions (93L, 51H) contacted the epitope solely through main-chain interactions, which were not affected by the mutated side-chains. Replacements at three other positions (31H, 54H, 56H) nearly perfectly recapitulated contacts, also conserving the interface. The 4E10 and GEP paratope/epitope interface is largely hydrophobic, thus binding is mainly mediated by Van der Waals contacts and desolvation entropy, which is conserved through equivalent positioning of nonpolar groups. This was reflected in the close concordance between the surface areas (SA) buried in the complexes by core epitope and the corresponding shape complementarity (Sc) [56] between Ab and core epitope. Only two somatic mutations were predicted to contribute to a stronger binding interaction. The Y/K32L mutation replaces a hydrogen bond involving the tyrosine hydroxyl with a salt bridge. The P/L95H mutation, involving non-contacting residues, restructured LCDR3 to reposition the side-chain of the conserved serine at 94L from a non-contacting position in GEPs to one contributing a hydrogen bond in 4E10 complex structures. However, the Y/K32L-P/L95H double mutation made on a GEP 1 background (GEP 1^m^) did not affect T_m_, only marginally increased affinity by about three-fold, and did not improve neutralization potency (**Figs. 2A, 2D** **,** **6** and **Table 1**), arguing that affinity and neutralization are largely influenced by somatic mutations through indirect effects beyond the ability to adopt the optimal binding conformer.

**Table 2:** Crystallographic data collection and refinement statistics.

	GEP 1	GEP 1/T117	GEP 2/T117	GEP 7	GEP 7/T117	1C6
**PDB accession code**	4LRN.pdb	4M8Q.pdb	4M62.pdb	4OB5	4ODX	4LCI.pdb
**Data collection** *						
Space group	*P*222	*P*2_1_	*P*2_1_2_1_2_1_	*P*2_1_2_1_2_1_	*P*2_1_	*P*2_1_2_1_2_1_
Cell dimensions						
*a*, *b*, *c* (Å)	35.54, 48.77, 110.7	72.56, 77.67, 77.82	69.88, 103.5, 109.3	35.54, 48.95, 117.27	72.91, 78.00, 78.40	44.60, 71.62, 73.23
α, β, γ (°)	90, 90, 90	90, 112.03, 90	90, 90, 90	90, 90, 90	90, 112.29, 90	90, 90, 90
Resolution (Å)	50.00-1.90 (1.93-1.90)	24.61-2.89 (2.95-2.89)	69.88-1.80 (1.86-1.80)	50.00-1.70 (1.73-1.70)	62.73-3.10 (3.21-3.10)	44.60–1.90 (1.97–1.90)
*R*_merge_	0.091 (0.434)	0.092 (0.428)	0.035 (0.149)	0.062 (0.232)	0.126 (0.421)	0.104 (0.255)
*I*/σ*I*	43.64 (6.46)	25.24 (2.82)	20.2 (5.00)	53.16 (8.63)	6.9 (1.9)	10.0 (4.1)
Completeness (%)	100.0 (100.0)	99.6 (97.8)	98.0 (91.7)	99.9 (100.0)	98.6 (97.6)	99.4 (95.0)
Redundancy	14.1 (14.3)	3.5 (3.0)	3.4 (2.4)	6.7 (6.6)	2.9 (2.9)	6.6 (4.4)
**Refinement**						
No. reflections	15,973 (753)	17,070 (861)	72,714 (6,712)	23,229 (1,127)	14,742 (1,437)	18024 (1,736)
*R*_work_/*R*_free_	0.190/0.233	0.261/0.302	0.189/0.216	0.178/0.207	0.280/0.304	0.179/0.230
No. atoms						
Protein	1,643	5,426	6,052	1,764	5,687	1854
Ligand/ion	–	–	45	23	–	25
Water	106	–	696	141	–	166
*B*-factors (Å^2^)						
Protein	25.34	59.85	19.41	26.09	56.94	28.86
Ligand/ion†	–	–	42.97	32.81	–	44.58
Water	33.90	–	27.26	35.61	–	36.47
R.m.s. deviations						
Bond lengths (Å)	0.004	0.004	0.003	0.014	0.004	0.015
Bond angles (°)	0.765	0.733	0.708	1.636	0.865	1.584
MolProbity score	1.19	1.01	0.83	1.34	1.48	1.16
Residues in most favored regions (%)	95.6	96.4	98.3	97.8	95.8	95.7
Res. in disallowed regions (%)	0	0	0	0	0	0
Est. coordinate error (max. likelihood ESUc) (Å)	0.108	0.428	0.079	0.113	0.555	0.107

*One crystal was used per data set. Values in parentheses are for the highest-resolution shell.

† The ligands identified in structures 4M62, 4OB5, and 4LCI are ordered sulfate ions and/or glycerol molecules. They do not affect the interactions between antigens and antibodies.

**Table 3:** Epitope contacts for 4E10 and GEPs determined from complex crystal structures; electrostatic contacts are **bolded**; otherwise, the contacts are mediated by van der Waals interactions.

NWFDIT epitope	4E10 Fab (16-mer peptide)	4E10 Fv (T88)	GEP 1 (T117)	GEP 2 (T117)	GEP 7 (T117)
Asn	–	–	L 32 TYR	L 32 TYR	L 32 TYR
	**L 91 TYR**	–	**L 91 TYR**	**L 91 TYR**	**L 91 TYR**
	L 91 TYR	–	L 91 TYR	L 91 TYR	L 91 TYR
	**L 92 GLY**	L 92 GLY	**L 92 GLY**	**L 92 GLY**	**L 92 GLY**
	L 92 GLY	L 93 GLN*	L 92 GLY	L 92 GLY	L 92 GLY
	L 93 GLN*	**L 94 SER**	L 93 SER*	L 93 SER*	L 93 SER*
	**L 94 SER**	L 94 SER	–	–	–
	L 94 SER		–	–	–
Trp	H 33 ALA	H 33 ALA	H 33 ALA	H 33 ALA	H 33 ALA
	H 50 GLY	H 50 GLY	H 50 GLY	H 50 GLY	H 50 GLY
	H 51 VAL*	H 51 VAL*	H 51 ILE*	H 51 ILE*	H 51 ILE*
	H 52 ILE	H 52 ILE	H 52 ILE	H 52 ILE	H 52 ILE
	H 56 ILE*	–	**H 56 THR**	–	–
	H 56 ILE*	H 56 ILE*	H 56 THR*	–	H 56 THR*
	–	**H 58 ASN**	–	–	–
	H 58 ASN	H 58 ASN	H 58 ASN	H 58 ASN	H 58 ASN
	**L 94 SER**	**L 94 SER**	–	–	–
	L 94 SER	L 94 SER	–	–	–
Phe	H 47 TRP	H 47 TRP	H 47 TRP	H 47 TRP	H 47 TRP
	H 100J PHE	H 100J PHE	H 100J PHE	H 100J PHE	H 100J PHE
	L 91 TYR	L 91 TYR	L 91 TYR	L 91 TYR	L 91 TYR
	L 93 GLN*	L 93 GLN*	L 93 SER*	L 93 SER*	L 93 SER*
	L 94 SER	L 94 SER	L 94 SER	L 94 SER	L 94 SER
	L 96 SER	L 96 SER	L 96 SER	L 96 SER	L 96 SER
Asp	L 32 LYS*	L 32 LYS*	L 32 TYR*	L 32 TYR*	L 32 TYR*
	L 32 LYS*	L 32 LYS*	L 32 TYR*	L 32 TYR*	L 32 TYR*
Ile	H 52 ILE	H 52 ILE	H 52 ILE	H 52 ILE	H 52 ILE
	H 54 LEU*	H 54 LEU*	H 54 PHE*	H 54 PHE*	H 54 PHE*
	H 56 ILE*	H 56 ILE*	H 56 THR*	–	H 56 THR*
Thr	H 31 THR*	H 31 THR*	H 31 SER*	H 31 SER*	H 31 SER*
	H 32 TYR	H 32 TYR	H 32 TYR	H 32 TYR	H 32 TYR
	H 33 ALA	H 33 ALA	H 33 ALA	H 33 ALA	H 33 ALA
	H 52 ILE	H 52 ILE	H 52 ILE	H 52 ILE	H 52 ILE
	**H 95 GLU**	**H 95 GLU**	**H 95 GLU**	**H 95 GLU**	**H 95 GLU**
	H 95 GLU	H 95 GLU	H 95 GLU	H 95 GLU	H 95 GLU
	H 100F PRO	H 100F PRO	H 100F PRO	H 100F PRO	H 100F PRO

Asterisks indicate residues that differ between GEPs and 4E10.

**Table 4:** Additional contacts between GEPs and the scaffold moiety of ES T117 outside of the grafted 4E10 epitope.

Epitope-scaffold T117	GEP 1	GEP 2	GEP 7
8 ALA	H 100E LYS	H 100E LYS	H 100E LYS
56 ARG	H 54 PHE	H 54 PHE	H 54 PHE
	H 55 GLY	H 55 GLY	H 55 GLY
	–	H 56 THR	H 56 THR
	–	H 56 THR	H 56 THR
60 PHE	H 54 PHE	H 54 PHE	H 54 PHE
	H 55 GLY	H 55 GLY	H 55 GLY
	–	H 56 THR	–
62 THR	H 54 PHE	H 54 PHE	H 54 PHE
64 LEU	H 53 ILE	H 53 ILE	H 53 ILE
73 ILE	H 54 PHE	H 54 PHE	H 54 PHE
75 HIS	H 54 PHE	H 54 PHE	H 54 PHE
100 ALA	H 53 ILE	H 53 ILE	H 53 ILE
	H 54 PHE	H 54 PHE	H 54 PHE
	A 73 LYS	H 73 LYS	H 73 LYS
101 GLY	H 73 LYS	H 73 LYS	H 73 LYS
125 ASN	H 100F PRO	H 100F PRO	H 100F PRO
	H 100E LYS	H 100E LYS	H 100E LYS
127 LEU	H 53 ILE	H 53 ILE	H 53 ILE
	H 54 PHE	H 54 PHE	H 54 PHE
128 TRP	–	H 100C LEU	–
	–	H 100C LEU	–
	H 100D GLY	H 100D GLY	H 100D GLY
	H 100E LYS	H 100E LYS	H 100E LYS
	H 100F PRO	H 100F PRO	H 100F PRO

### GEP structures revealed that structural plasticity was retained during affinity maturation

**Figure 9:**
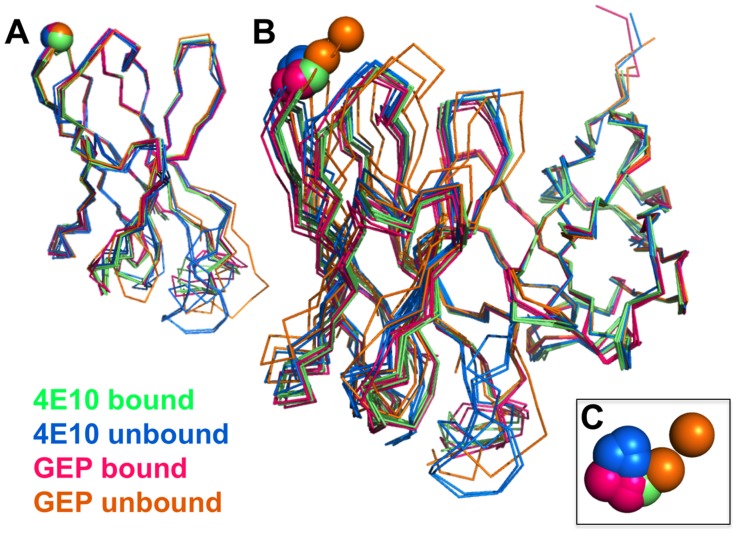
Interdomain movements within Fv cassettes are limited. (**A**) Superpositions of the V_H_ domains from two 4E10 ligand-bound structures (2FX7.pdb, 3LH2.pdb), unbound 4E10 (4LLV.pdb), ligand-bound GEP 1 (4M8Q.pdb), unbound GEP 1 (4LRN.pdb), ligand-bound GEP 2 (4M62.pdb), ligand-bound GEP 7 (4ODX.pdb), and unbound GEP 7 (4OB5.pdb) are shown in Cα backbone representations, colored as indicated. Residue P14H in each Fv, chosen as a reference point to illustrate interdomain movement upon binding between 4E10 and GEPs, is shown as a sphere and colored to match the corresponding backbone. When isolated V_H_ domains are superimposed, the P14H spheres are nearly coincident, indicating that the V_H_ domain structure is highly conserved among these Abs. (**B**) Fv cassettes of 4E10 and GEPs, colored as in (**A**), superimposed only on V_L_ domains (oriented on the right side of the panel), are shown as represented in (**A**). In this view, interdomain movements can be visualized as the relative movement of V_H_ domains, particularly at the P14H reference point. (**C**) Orthogonal view of the P14H spheres excerpted from (**B**) illustrating the pattern and degree of interdomain movements.

**Figure 10:**
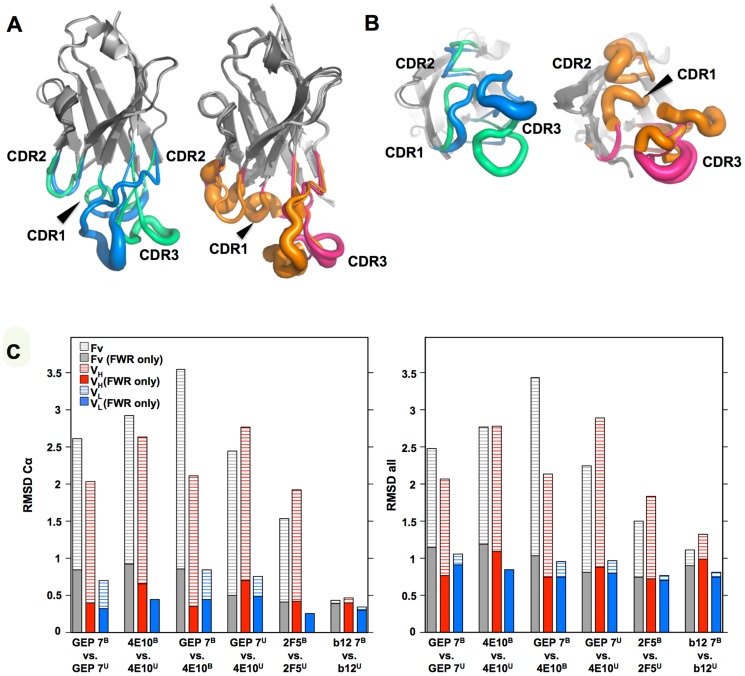
CDR restructuring. Views from the side (A) and below (B) are shown of superpositions of isolated V_H_ domains, in cartoon representations, with FRW regions colored grey and β-strands indicated as arrows. HCDRs are shown in a B-factor putty representation (tube width correlates with crystallographic Debye-Waller, or B, factor), colored by Ab. On the left in both frames is shown the superposition of V_H_ domains from ligand-bound 4E10 (3LH2.pdb, with green HCDRs) vs. unbound 4E10 (4LLV.pdb, with blue HCDRs), and on the right in both frames is shown the superposition of ligand-bound structures of GEP 1 (4M8Q.pdb), GEP 2 (4M62.pdb), and GEP 7 (4ODX.pdb), all with pink HCDRs, vs. unbound GEP 1 (4LRN.pdb) and GEP 7 (4OB5.pdb), both with orange HCDRs. (**C**) RMSD values for V_H_, V_L_, or Fv superpositions, calculated with or without CDR residues, are plotted for Cα atoms only (*left*) or all atoms (*right*).

In order to determine whether the 4E10 combining site rigidified during affinity maturation, undergoing binding site preconfiguration, the crystal structures of GEP 7 and GEP 1 were determined in the unbound state at resolution values of 1.9 Å and 1.7 Å respectively, rebuilt and refined with good statistics (**Table 2**), and compared to the unbound structure of 4E10 (4LLV.pdb [17]). Superpositions of 4E10 and GEP bound and unbound structures showed that interdomain movements, while present, were limited compared to CDR rearrangements (**Fig. 9**), and that 4E10 retained at least as much CDR flexibility as was present in GEPs, particularly in HCDR2 and HCDR3, while LCDRs were relatively constrained (**Figs. 10A and 10B**). Calculated root mean square deviations (RMSDs; **Fig. 10C**) of V_H_ and V_L_ superpositions, with and without CDRs, confirmed that while the FWR regions of GEPs and 4E10 were nearly identical, the bulk of rearrangements observed during binding occurred in HCDRs, with 4E10 movements as large, or larger, than observed in GEPs. LCDR movements, while much smaller overall, contributed less to rearrangements in 4E10 than GEPs, suggesting some minimal degree of rigidification during maturation. Comparison of the surfaces directly contacting epitopes (**Movies S1** and **S2**) showed that GEPs retained higher degrees of structural conservation than 4E10 between the bound and unbound states. These analyses need to be interpreted cognizant of the constraints imposed by crystallization, which involved varying non-physiological solution conditions and variable crystal contacts between structures. However, these caveats were minimized by comparing multiple structures with multiple copies per asymmetric unit.

### Engineered ESs can make extensive contacts outside of the targeted epitope

**Figure 11:**
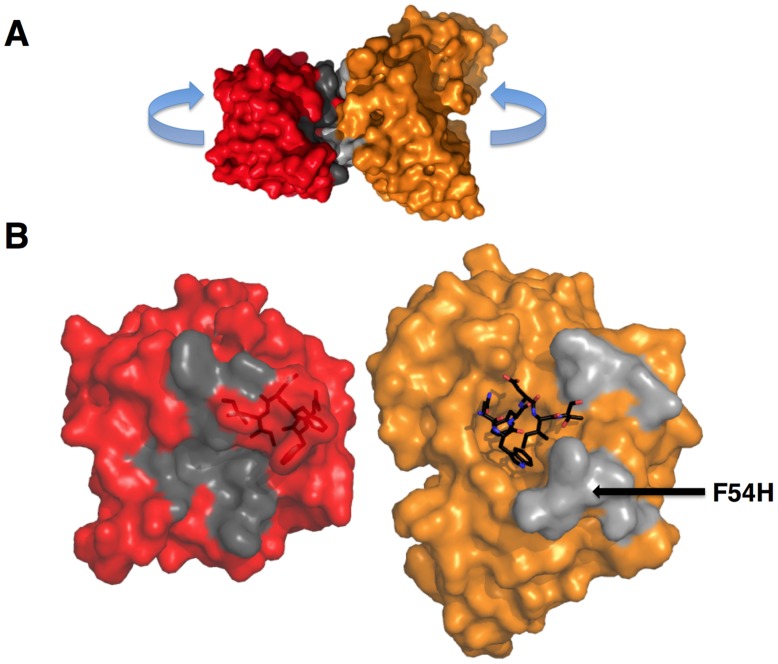
GEPs binding surfaces on T117. (**A**) The complex of GEP 2 (orange) bound to ES T117 (red) is shown in molecular surface representations. Non-epitope scaffold contacts are highlighted in grey. (**B**) The interaction partners were separated as indicated in (**A**) to reveal details of the interface. T117, on the left, is shown in a red semi-transparent molecular surface representation with the NWFDIT epitope, in a licorice-stick representation, visible within the surface. The GEP 2 Fv structure is shown on the right, colored as in (**A**). The NWFDIT epitope, excised from the T117 structure, is shown as bound for reference. The footprint of GEP 2 (non-epitope contacts) on T117 is colored dark grey; the footprint of T117 to non-epitope residues on GEP 2 is colored light grey. The position of the side-chain of F54H is indicated.

The goal of reverse engineering an Ab is to scaffold the desired epitope to re-elicit Abs that solely recognize the epitope [6], [57]. However, the 4E10 linear epitope, as currently defined, is smaller than typical Ab/antigen interfaces, which poses the design challenge of isolating humoral responses to the epitope and not contiguous scaffold surfaces. Previous crystal structures of 4E10/ES complexes showed that many ESs achieved this goal well, including T93 [15], [55]. However, GEP/T117 complex structures reported here showed extensive GEP/scaffold contacts (SA for GEP2 contacts to scaffold minus epitope = 308 Å^2^) (**Fig. 11**). A dominant feature of these interactions was the binding of the side-chain of the GEP-specific residue F54H in a deep hydrophobic cleft of the T117 scaffold protein, a putative phosphotransferase from *S. typhimurium*. These additional contacts raise the concern that Abs elicited by T117 immunizations may have off-target (non-Env) specificities. Nevertheless, the T117 scaffold is highly complementary to 4E10 (Sc for GEP2/T117 = 0.69), which may help explain the increased affinity of T117 for 4E10 and GEPs, and may be ideal for preferentially targeting GEPs through the F54H interaction (F54H is present in the heavy chain gene used by all GEPs). The additional T117 contacts appear to have had the effect of increasing the affinity for T117 over other ESs by two orders of magnitude for 4E10 and by one to three orders of magnitude for the GEPs. However, structural superpositions show that these extra Ab/scaffold interactions do not affect Ab/epitope interactions, or the structure of the epitope in the complexes, which are highly conserved (**Fig. 8B**).

### GEPs displayed a similar degree of limited polyspecificity to 4E10, but different autoreactivity

**Figure 12:**
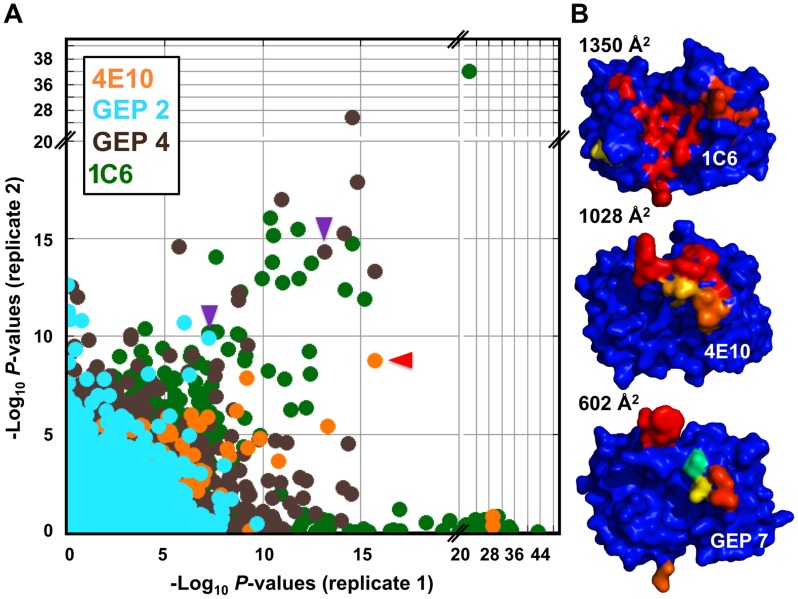
Peptidome binding results. (**A**) PhIP-Seq results are plotted as −Log_10_ *P*-values, one replicate on the abscissa, the other on the ordinate, colored by Ab as indicated; note the discontinuity in axis scales. The top scoring 4E10 peptide derived from IP_3_R is highlighted with a red arrow; one peptide, derived from the zinc finger Ran-binding domain-containing protein 3, which bound to both GEP 2 and GEP 4, is highlighted with purple arrows. Proximity to the diagonal indicates good replicate concordance; peptides with highly discordant replicate values, falling along the axes, were discarded from the analysis. Overall library scoring statistics are: 4E10, average = 0.32, σ = 0.35; GEP 2, average = 0.22, σ = 0.44; GEP 4, average = 0.25, σ = 0.52; 1C6, average = 0.27, σ = 0.50. (**B**) The molecular surfaces of the Fv domains of 1C6 (4LCI.pdb), unbound 4E10 (4LLV.pdb) and unbound GEP 7 (4OB5.pdb) are shown, oriented with V_H_ domains at left and the V_L_ domains at right. The surface is colored to show hydrophobic patches, defined by the program HotPatch [80]; patches are colored in descending order of total area (red, orange, yellow, …). The total surface area for red and orange hydrophobic patches is shown. The crystal structure of GEP 7 is partially disordered in HCDR1 and 3 and so patch area is underrepresented in the calculation.

A validated phage-displayed library consisting of 413,611 overlapping 36-mer peptides spanning the entire human proteome combined with phage immunoprecipitation sequencing (PhIP-Seq) was used to assess the polyspecificity and autoantigen recognition profiles of GEPs in comparison to 4E10 [17], [50] (**Fig. 12A**, **Tables 5**, **6**, and **7**). GEP 2 and GEP 4 were selected to represent both the clustering (GEP 2) and 96H mutant (GEP 4) GEPs, and an affinity-matured, murine anti-canine CD28 Ab, 1C6 [58], was included for comparison (**Fig. 12A**, **Table 8**). The top hit in the 4E10 Fv PhIP-Seq analysis reported here, a peptide derived from the type 2 inositol 1,4,5-trisphosphate receptor (IP_3_R), matches the top hit from the previous PhIP-Seq analysis of IgG 4E10 [17]. However, overall scores were considerably reduced in the PhIP-Seq analysis of 4E10 as an Fv construct versus intact IgG (replicate average −Log_10_ *P*-values for the top ten scoring peptides of 35.3 to 255 for IgG versus 4.35 to 12.3 for Fv), likely due to decreased accessibility of coupled Fv relative to IgG, an increased chance of inactivating Fv versus IgG during chemical coupling, and the inherent increase in local avidity of bivalent IgG versus univalent Fv on potentially sparsely-coupled beads. Given these caveats, overall scoring behavior was very similar across the Fvs tested, with 4E10 and 1C6, the affinity-matured Abs, showing the highest average scores. GEP 4 and 1C6 showed the largest number of high-scoring hits, with 61 and 194 peptides with replicate-averaged −Log_10_ *P*-values of ≥4.0 respectively; 4E10 and GEP2 had 12 and 20 peptides, respectively, scoring ≥4.0. Qualitatively, the results were not dramatically different, but with GEP 4 and 1C6 showing nominally greater spreads of top-scoring peptides rising above the bulk responses. None of the top-scoring three-dozen 4E10 peptides appeared in the top three-dozen hits from either GEP; however two of the top-scoring dozen GEP peptides (derived from zinc finger Ran-binding domain-containing protein 3 or hyaluronidase-3 isoform 1 precursor) were in common between GEP 2 and GEP 4, scoring 1 and 12 (GEP 2) and 4 and 2 (GEP 4), respectively. However, no common peptide motifs could be identified within or between Fv results. None of the IP_3_R peptides scored in the top 65,000 GEP hits. While 1C6 showed the highest scoring spread of top hits (replicate average −Log_10_ *P*-values for the top ten scoring peptides of 12.14 to 30.9), the top ten scoring peptides displayed a considerably higher average hydrophobic character (Φ), with average Φ values of: 4E10 = 0.37; GEP 2 = 0.42; GEP 4 = 0.45; 1C6 = 0.71 (higher values are more hydrophobic). Using relative hydrophobicity of the top-scoring PhIP-Seq peptides as a surrogate measure of the overall hydrophobicity of the combining site was consistent with the structures of the Abs (**Fig. 12B**), where the 1C6 combining site is structured as a large, broad, very hydrophobic concavity and 4E10 and its GEPs sporting smaller, convex hydrophobic surfaces.

**Table 5:** PhIP-Seq results for 4E10 Fv.

Rank	Avg. −log*P*	Peptide sequence	Peptide source	Φ	Charge
1	12.25	SKCRVFNTTERDEQGSKV	inositol 1,4,5-trisphosphate receptor type 2	0.17	−1
		NDFFQQTEDLYNEMKWQK			
2	9.37	PPPRCISTNKCTAPEVE	complement receptor type 1 isoform S precursor	0.48	+1.9
		NAIRVPGNRSFFSLTEIVR			
3	7.32	KAFNYRSYLTTHQRSHTG	zinc finger protein 267	0.18	+4.1
		ERPYKCEECGKAFNSRSY			
4	7.22	PPHELTEEEKQQILHSE	cytoplasmic dynein 1 intermediate chain 2	0.28	−5.7
		EFLSFFDHSTRIVERALSE			
5	6.22	AHTGEKPYVCRECGRGFR	Krueppel-related zinc finger protein 1	0.10	+5.4
		QHSHLVRHKRTHSGEKPY			
6	5.21	STPIEWYPDYEVEAYRRR	XP_373395.2	0.49	−1.7
		HHNSSLNFFNWFSDHNFA			
7	4.72	EQGIVGPRWWVFPSLRFAA	XP_498650.1	0.71	+2
		VSRPFCGAWVLSWGQAT			
8	4.49	GAQPPFDAQSPLDSQPQPS	nuclear fragile X mental retardation-interacting protein 1	0.42	−0.9
		GQPWNFHASTSWYWRQS			
9	4.36	LYEEISMPLLADVRLNYLG	inter-α-trypsin inhibitor heavy chain H6 precursor	0.74	−2
		GLVGASPWAVFPNYFGG			
10	4.25	YKVDVTWTRARGASRGWR	TP53-target gene 5 protein	0.12	+10.1
		SRHQLKGRNGWRNSRVYK			
11	4.15	AKWREVSHTFSNYPPGVR	F-box only protein 44 isoform 1	0.58	+1.3
		YIWFQHGGVDTHYWAGWY			
12	4.07	MNPQIRNPMKAMYPGTFY	APOBEC-3C	0.61	+1
		FQFKNLWEANDRNETWLC			
13	3.78	SGGYGSGGYGGSATPSGR	ATP-dep. RNA helicase A	0.22	+4
		ICAGVGGGYRGVSRGGFR			
14	3.77	LMMKKRVRLEEAFEFVK	dual specificity protein phosphatase 4 isoform 2	0.48	+4
		QRRSIISPNFSFMGQLLQF			
15	3.59	DGMYQRFLRQHVHPEETG	ribonuclease 4 precursor	0.28	+2.1
		GSDRYCNLMMQRRKMTLY			
16	3.47	TSLEVEPFASLTEAVRSS	NAD-dependent deacetylase sirtuin-3, mitochondrial isoform a	0.56	−0.9
		VPRLLINRDLVGPLAWHP			
17	3.40	SEQFYDRSLGIMRRVLPP	secreted phosphoprotein 24	0.26	+5.2
		GNRRYPNHRHRARINTDF			
18	3.38	MKPGFSPRGGGFGGRGGF	rRNA 2′-O-methyltransferase fibrillarin	0.03	+7
		GDRGGRGGRGGFGGGRGR			
19	3.31	NARRFSAGQWEARRGWRL	ribosomal biogenesis protein LAS1L isoform 1	0.43	+2.9
		FNCSASLDWPRMVESCLG			
20	3.23	ESWLSRFSYAWLAPLLAR	multidrug resistance-associated protein 7 isoform MRP7A	0.51	+2
		GACGELRQPQDICRLPHR			

**Table 6:** PhIP-Seq results for GEP 2 Fv.

Rank	Avg. log*P*	Peptide sequence	Peptide source	Φ	Charge
1	8.35	EGWQCSLCTYINNSELPY	**zinc finger Ran-binding domain-containing protein 3**	0.61	−5.2
		CEMCETPQGSAVMQIDSL			
2	6.11	PVFSFSKTSEYHDIMYPA	protein O-glucosyltransferase 1 precursor	0.72	−1.9
		WTFWEGGPAVWPIYPTGL			
3	5.74	KVTDTKPRVAEWRYGPAR	transcription initiation factor TFIID subunit 1-like	0.29	−1
		LWYDMLGVSEDGSGFDYG			
4	5.58	MTTFTEPEVVFLQSRGNE	arf-GAP domain and FG repeat-containing protein 2	0.56	0
		VCRKIWLGLFDARTSLVP			
5	5.57	SGWGSRSQAPYGTLGAVS	peroxisome proliferator-activated receptor γ coactivator-related protein 1	0.32	−2.9
		GGEQVLLHEEAGDSGFVS			
6	5.05	MGLSRRNPSYPWLWEDG	oxidized low-density lipoprotein receptor 1 isoform 1	0.53	+2.1
		SPLMPHLFRVRGAVSQTYP			
7	4.90	ASTLGSMPSFTARLTRGQL	RING finger protein 37 isoform a	0.33	+4.1
		QHLGTRGSNTSWRPGTG			
8	4.72	QPNPHGNMMYTGPSHHSY	histone acetyltransferase KAT6A	0.40	+3.3
		MNAAGVPKQSLNGPYMRR			
9	4.62	KLMGKDESTSRNRRSLSP	rho guanine nucleotide exchange factor 18 isoform a	0.25	+3.1
		ILPGRHSPAPPPDPGFPA			
10	4.47	IQRYCNCNSSMPRPVKVA	phosphofurin acidic cluster sorting protein 1	0.54	+4.9
		AVGGQSYLSSILRFFVKS			
11	4.44	RYDVQERHPKGKMIPVLH	protein phosphatase 1 regulatory subunit 17 isoform 1	0.09	+4.3
		NTDLEQKKPRRKDTPALH			
12	4.39	RPGFAGPAVLDWEEWCPL	**hyaluronidase-3 isoform 1 precursor**	0.58	+1
		WAGNWGRRRAYQAASWAW			
13	4.39	PIPSGSYYAPYGMPYTSM	SET-binding protein isoform a	0.71	0
		PMMNLGYYGQYPAPLYLS			
14	4.28	NQSQGCLPARTCHSPAHS	thrombospondin-3 isoform 1 precursor	0.50	+1.2
		PCHIHAHCLFERNGAVSC			
15	4.27	LEREVTDVDSVVGRSSVG	dedicator of cytokinesis protein 8 isoform 1	0.11	+1
		ERRTLAQSRRLSERALSL			
16	4.23	RSRRKQHLLPPCVDEPEL	forkhead box protein M1 isoform 1	0.38	+0.1
		LFSEGPSTSRWAAELPFP			
17	4.22	TQAFDFYSRYFAPWVGVA	phenazine biosynthesis-like domain-containing protein, isoform a	0.53	−1.9
		EDPVTGSAHAVLSSYWSQ			
18	4.17	TKWMNMKAVFGHPFSLGW	palmitoyltransferase ZDHHC3 isoform 2	0.52	+1.1
		ASPFATPDQGKADPYQYV			
19	4.11	AGSWHPRSYAAYALKTW	kiSS-1 receptor	0.68	+2.1
		AHCMSYSNSALNPLLYAFL			
20	4.07	EETAGEPWEDGFEAELSP	NP_955358.1	0.31	−5
		VEQKLSALRSPLAQRPFF			

**Table 7:** PhIP-Seq results for GEP 4 Fv.

Rank	Avg. −log*P*	Peptide sequence	Peptide source	Φ	Charge
1	16.36	PKGKCLGSQDYLELANRF	S phase cyclin A-associated protein in the ER isoform a	0.21	+3
		PQQAWEEARQFFLKKEKK			
2	14.52	RPGFAGPAVLDWEEWCPL	**hyaluronidase-3 isoform 1 precursor**	0.58	+1
		WAGNWGRRRAYQAASWAW			
3	13.98	ISHIQPFSFLDLESLRSL	leucine-rich repeat and fibronectin type-III domain-containing protein 2 precursor	0.50	−1.8
		HLDSNRLPSLGEDTLRGL			
4	13.73	EGWQCSLCTYINNSELPY	**zinc finger Ran-binding domain-containing protein 3**	0.61	−5.2
		CEMCETPQGSAVMQIDSL			
5	9.43	LEQNLAAAEEGPLEPAVV	NP_957704.1	0.59	−5.9
		DAFNQAWHLFAHECPNYF			
6	9.40	QAAPAPQPVFVGPAVPQG	Krueppel-like factor 11 isoform a	0.68	0
		AVMLVLPQGALPPPAPCA			
7	8.16	EETAGEPWEDGFEAELSP	NP_955358.1	0.31	−5
		VEQKLSALRSPLAQRPFF			
8	7.40	SEALSRDPETLVGYSMVG	fatty acid synthase	0.45	0
		CQRAMMANRLSFFFDFRG			
9	7.37	RKREKCWGRSSVMAEYG	visual system homeobox 2	0.34	+4
		LYGAMVRHSIPLPESILKS			
10	6.65	LIKTKQRKESRFQLFMQ	rho guanine nucleotide exchange factor 11 isoform 1	0.27	+4
		EAESHPQCRRLQLRDLIIS			
11	6.06	SDSSWQQQPGQPPPHSTW	uncharacterized protein C14orf43	0.38	+0.2
		NCHSLSLYSATKGSPHPG			
12	5.98	NGKIKYECNVCAKTFGQL	PR domain zinc finger protein 1 isoform 2	0.40	+5
		SNLKVHLRVHSGERPFKC			
13	5.97	KGKAGTPSGSSADEDTF	suppressor of cytokine signaling 6	0.11	+2
		SSSSAPIVFKDVRAQRPIR			
14	5.75	DEMKEIQERQRDKLYERR	transcriptional activator protein Pur-β	−0.25	−8
		GGGSGGGEESEGEEVDED			
15	5.64	QPPFFSKEQPQALNFGGI	membrane metallo-endopeptidase-like 1	0.44	−1.8
		GMVIGHEITHGFDDNGRN			
16	5.60	RTTENPTLERKPYSSPR	zinc finger FYVE domain-containing protein 26	0.23	+3
		DSSLPALTSSALAFLKSRS			
17	5.60	NQSQGCLPARTCHSPAHS	thrombospondin-3 isoform 1 precursor	0.50	+1.2
		PCHIHAHCLFERNGAVSC			
18	5.29	MAKKGCRHLVCSSGGNA	serine dehydratase-like	0.48	+5
		GIAAAYAARKLGIPATIVL			
19	5.28	TTPFTLEGRPRGELHEQY	dedicator of cytokinesis protein 8 isoform 1	0.33	+3.2
		RRNTVLTTMHAFPYIKTR			
20	5.26	MSAQSLPAATPPTQKPPR	rho guanine nucleotide exchange factor 15	0.25	+6
		IIRPRPPSRSRAAQSPGP			

**Table 8:** PhIP-Seq results for 1C6 Fv.

Rank	Avg. −log*P*	Peptide sequence	Peptide source	Φ	Charge
1	30.87	AVLKYENNVMNIRQFNCS	Ser/Thr-protein phosphatase 2B catalytic subunit α isoform 1	0.81	0
		PHPYWLPNFMDVFTWSLP			
2	14.67	PTWDQVPPFQWSTSPFSG	putative phospholipase B-like 2 isoform 1	0.64	+0.1
		LLHMGQPDLWKFAPVKVS			
3	13.63	QGLVLNWGLMTTRGQGLM	*Record removed* (XP_495971.1)	0.70	+0.1
		SSWGLGAHWGLPVNLGTG			
4	13.54	AGNHFINVMLSHPNHTGN	trans-2,3-enoyl-CoA reductase-like	0.86	+0.2
		NACFPSPNYNPFTWMFFL			
5	13.30	PIPSGSYYAPYGMPYTSM	SET-binding protein isoform a	0.71	0
		PMMNLGYYGQYPAPLYLS			
6	13.21	MASNSSSCPTPGGGHLNG	retinoic acid receptor α isoform 1	0.69	0
		YPVPPYAFFFPPMLGGLS			
7	13.11	MNPQIRNPMKAMYPGTFY	APOBEC-3C	0.61	+1
		FQFKNLWEANDRNETWLC			
8	12.85	PENRGGFQGFGFGDGGF	E3 ubiquitin-protein ligase RNF185 isoform 1	0.61	−1
		QMSFGIGAFPFGIFATAFN			
9	12.41	MNHMLPDPGTWEEYFETFI	sulfotransferase 1C2 isoform a	0.70	−2.8
		NGKVVWGSWFDHVKGWW			
10	12.14	LQPPPSRFKQFFCLSLPS	WD repeat-containing protein 27 isoform 1	0.73	+1
		SWDYSLPQLPWMVNSSSF			
11	11.87	TDRYMWSDASGLQECTKA	tectonin β-propeller repeat-containing protein 1	0.45	−2
		GTKPPSLQWAWVSDWFVD			
12	11.46	EYGPPRKQPKQQHGPGFW	uncharacterized protein C12orf12	0.52	+3
		FQPPVCSNWGCWGGPWRP			
13	10.84	KVTDTKPRVAEWRYGPAR	transcription init. factor TFIID subunit 1-like	0.29	−1
		LWYDMLGVSEDGSGFDYG			
14	10.80	EQGKEPWMVVREETGRWC	zinc finger protein 461	0.45	−1.9
		PGTWKTWGFHNNFLDNNE			
15	10.57	TYGEEGLKDGHQSSHGDI	dnaJ homolog subfamily B member 11 precursor	0.33	−2.7
		FSHFFGDFGFMFGGTPRQ			
16	10.26	FWGTGLSLPSLPVSFPLQ	metallothionein-1E	0.75	+2
		AFCPKFRWGRTAFFSWDT			
17	9.46	PVFSFSKTSEYHDIMYPA	protein O-glucosyltransferase 1	0.72	−1.9
		WTFWEGGPAVWPIYPTGL			
18	9.45	NTTWYSNDTWYGNDTWYG	sodium channel protein type 4 subunit α	0.60	−5
		NEMWYGNDSWYANDTWNS			
19	9.41	STRLPSEYIYGFGEVEHTA	sucrase-isomaltase, intestinal	0.42	−0.9
		FKRDLNWNTWGMFTRDQ			
20	9.27	SQWGQWSQVYGNPQQYGQ	nucleolysin TIAR isoform 1	0.59	0
		YMANGWQVPPYGVYGQPW			

### Pre-binding of 4E10 at the MPER affects the binding of bnAb b12 at the CD4 binding site

**Figure 13:**
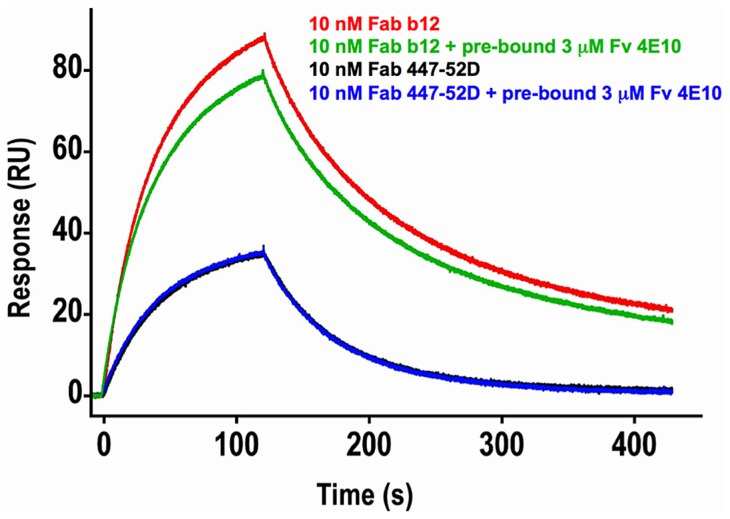
Effects of 4E10/gp140_3_ pre-binding on 447-52D and b12 binding. Double-corrected sensorgrams of the SPR response of 10 nM analytes of Fab b12 (red and green traces) or Fab 447-52D (blue and black traces) to chip-coupled SF162 gp140_3_ in the absence (red and black traces) or presence (green and blue traces) of pre-bound Fv 4E10 are shown. Fv 4E10, at a concentration of 3 µM, was flowed over the chip starting 5 minutes before the 447 or b12 injections and continued throughout the experiment. The black and blue 447-52D traces were completely superimposed, showing no effect of 4E10 pre-binding on 447-52D binding. While the dissociation phases of the b12 traces are nearly parallel, showing no minimal effect of 4E10 pre-binding on the dissociation of b12 from the bound state, the association phases are clearly not parallel, resulting in a lower peak response in the presence of 4E10 pre-binding, showing a modest, but important, reduction in the association rate. This result suggests that the CD4 binding site adopted a distinct conformational state prior to b12 binding as a consequence of 4E10 binding at the MPER.

In order to test the hypothesis that 4E10 may induce global conformational changes in gp140_3_ as part of a higher-order neutralization mechanism [17], [21], consistent with downstream effects such as gp120 release, binding of Abs to epitopes distant from the MPER (the V3 loop, by 447-52D [59], and the CD4 binding site, by b12 [60]) was assayed by SPR in the presence or absence of saturating 4E10 (**Fig. 13**). The qualitative results show that 4E10 pre-binding does not affect 447-52D binding to the flexible, extended V3 loop, but does alter the association kinetics of b12 at the CD4 binding site, suggesting that 4E10 binding induced global conformational changes in gp140_3_ registering at distant sites. Since b12 dissociation kinetics were unaffected, 4E10 did not preclude achieving a similar b12 bound-state conformation.

## Discussion

Reverse engineered ESs are ideal reagents for studying the binding properties of GEPs. Where peptides and Env do not display sufficient affinities, the best ESs display strong affinities across the GEP ensemble, allowing for the biophysical characterization of 4E10 ontogeny by using ESs to compare 4E10 and GEP binding kinetics and bound-state crystal structures. Consistent with current theory and previous results comparing GEPs with matured bnAbs or other Abs, 4E10 displays orders-of-magnitude better affinities than candidate GEPs for both Env gp140_3_ proteins and engineered ESs. This gain in affinity is potentially sufficient to account for the concurrent gain in neutralization potency over GEPs. Previous results showing minimal contributions from FWR mutations [61] and comparisons of complex structures of 4E10 with GEP/T117 complex structures, which showed near-complete conservation of the recognition interface (**Fig. 8**), argue that affinity maturation was likely due, at least in part, to the small number of somatic mutations in the CDR regions, *e.g.* 32L and 95H, that add or improve direct contacts to epitope. This limited number of mutations is presumably readily achievable during conventional vaccination. Based on PhIP-Seq peptidome binding results (**Fig. 12A**), 4E10 and GEPs have distinct autoantigen profiles, suggesting that 4E10 autoreactivity was acquired during ontogeny and not inherent in GEPs, consistent with recognizable GEPs populating naïve repertoires of mature B cells at relatively high frequencies [16]. Among ESs, T117 in particular interacts with GEPs sufficiently strongly to drive B cell activation and maturation (**Fig. 2D**) and selectively interacts with GEP-specific structural features, *e.g.* F54H (**Fig. 11**). These aspects of 4E10 combine to seemingly argue that 4E10 ontogeny follows a relatively short path and that directed re-elicitation of 4E10, and perhaps other bnAbs, may be an achievable goal.

In many aspects, however, 4E10 ontogeny appears more convoluted. Contrasting with the consensus model of Ab ontogeny and results from other systems [38], 4E10 is considerably less thermostable than GEPs, which was difficult to account for only from static views of structures. This lowers the headroom available to tolerate mutations that increase affinity or potency that otherwise might degrade stability, potentially adding a significant hurdle to re-elicitation of 4E10, but clearly does not limit the ability of 4E10 to bind to or neutralize HIV. The conformer ensemble sampled by GEP combining sites echoes the startling plasticity of 4E10, with structural comparisons of bound versus unbound states (**Figs. 9** and **10**) showing perhaps a small degree of 4E10 LCDR rigidification, but an increase in the conformer ensemble sampled by the HCDRs. This directly contradicts the consensus model, and specific examples [40], of Ab maturation, showing alternatively that preconfiguration of 4E10 does not occur during, or contribute to, affinity maturation. HCDR flexibility is highlighted by F29H, which is able to flip out into solvent in the unbound 4E10 and GEP 1 structures, allowing HCDR1 to dynamically sample multiple conformers. A high number of conserved glycine residues may contribute to 4E10 and GEP HCDR mobility. The reduced affinities due to decreased *k*_a_s (**Fig. 2D**), relative to the clustering GEPs, of GEP 4 and 6 with non-glycine residues at position 96H, predicted to restrict HCDR3 conformer sampling, suggests that HCDR3 mobility was needed to achieve the bound-state conformation with optimal kinetics by destabilizing non-optimal conformers. Retention of significant combining site plasticity also strongly argues for a functional role other than polyspecificity, which is unremarkable in comparison to other Abs, based on PhIP-Seq peptidome binding results (**Fig. 12A**). Minimal focusing of apparent polyspecificity also contradicts the consensus model, and specific examples [62], of maturation. Also unexpectedly, the ∼100-fold increase in affinity of 4E10 over GEPs to T117 is through a decreased *k*_d_ (**Fig. 2D**), suggesting that improvements in binding affinity arose from a more favorable bound state and not a decrease in entropic barriers imposed by more plastic GEPs. Thermodynamic studies of 4E10 and GEPs were not possible because no single antigen, needed for valid comparisons, bound to both 4E10 and GEPs with parameters accessible to measurement. However, while crystal structures showed a highly-conserved bound state in both 4E10 and GEPs, the small number of improved contacts could not account fully for the observed improvement in affinity. Complicating this analysis, and its use as a vaccine immunogen, are the extensive contacts between GEPs, and presumably also 4E10, and the scaffold of T117 outside of the stabilized MPER epitope (**Fig. 11**). The interaction with T117 also highlights another possibility. The 4E10 combining site extends beyond the minimal, linear epitope in T117, making contacts to the scaffold. Unless the MPER epitope forms an isolated structure extended away from the rest of Env, 4E10 may make contacts to elements of Env outside of the linear 4E10 epitope, as it does with T117. HCDR conformer dynamics may therefore be understood as enabling such interactions, which may foster conformational changes in Env leading to neutralization by any of several mechanisms. Prebinding of 4E10 to gp140_3_ affected b12 binding at the CD4 site distant from the MPER (**Fig. 13**), indirectly demonstrating a global conformational change consistent with this supposition. Additional interactions between GEPs and the scaffold moiety of T117 would also be predicted to drive rigidification of an even greater portion of the combining site during conventional immunization, increasing the distinction between 4E10 and a matured Ab derived from a 4E10 GEP stimulated with T117.

T117 was the product of a cutting-edge design effort to generate a structurally-stabilized MPER epitope with optimized binding properties for use as a vaccine immunogen to drive 4E10 ontogeny based on the current paradigm of affinity maturation. However, 4E10 appears to take the affinity maturation pathway less traveled, one that contrasts, in many fundamental ways, the current paradigm. This raises the concern that conventional immunization protocols, based on the current paradigm of structural stabilization of optimal binding conformers, will not successfully re-elicit 4E10 or other unconventional Abs. Understanding unconventional maturation pathways then becomes paramount for the future of molecular vaccinology, allowing efforts to focus on re-eliciting Abs identified as the products of conventional ontogeny in the near term while developing unconventional vaccination strategies to target exceptional Abs in the long term.

## Materials and Methods

### Protein prediction, expression, purification and characterization

The 4E10 heavy and light chain nucleotide sequences were analyzed using JoinSolver [63], IMGT/V-QUEST [64], [65], Ab-Origin [66], SoDA [67] and iHMMune [68] to compositely identify segments with the fewest nucleotide mismatches, generating a combinatorial ensemble of 12 GEPs (**Fig. 1**). Each derived GEP candidate consisted of a single V_L_ domain sequence assigned with high confidence (IGKV3-20*01 plus IGKJ1*01), one V_H_ gene segment also confidently assigned (IGHV1-69*06), plus one of six likely D segments (IGHD1-1*01, IGHD6-19*01, IGHD7-27*01, IGHD6-25*01, IGHD1-26*01, IGHD4-17*01) and either of two likely J segments (IGHJ4*02, IGHJ1*01). The CDR3 junctions and presumed N-nucleotide insertions in mature 4E10 were retained in the candidate GEPs. Sequences are numbered following Kabat [24]. 4E10, eight of the 12 GEPs, and 1C6 Fvs were engineered, expressed as inclusion bodies in *E. coli* BL21(DE3) RIL cells (Invitrogen), refolded, purified, and validated by SEC and PAGE as previously described [18]. Circular dichroism (CD) spectra of 4E10 Fv and GEPs were measured on a J-815 spectrometer (Jasco) at a concentration of 10 µM in 10 mM Na_2_HPO_4_/KH_2_PO_4_ (pH = 7.4). Temperature melts were performed at 210 nM with a temperature ramp from 25°C to 95°C at a slope of 2°C/minute, data pitch of 2°C, and delay time of 10 s (**Fig. 2**). T_m_s were determined by nonlinear least-squares analysis using a linear extrapolation model with Spectra Analysis software (Jasco). Fabs of 447-52D and 4E10 were prepared by digestion of IgG with papain (Pierce), affinity chromatography with protein A (Pierce) and preparative SEC (Superdex 200 16/10 column; GE Healthcare); IgG 4E10 was purchased from Polymun Scientific, and IgG 447-52D and Fab b12 were kindly provided by Pamela Bjorkman (Caltech).

### Neutralization assays

Neutralization assays (**Fig. 2B**) were performed using single-round entry-competent viruses and TZM-bl cells as previously described [53]. Percent neutralization at concentrations of 0.96 µM (4E10 and GEP Fvs) or 0.17 µM (4E10 IgG) was calculated as previously described [18], [53].

### SPR interaction analyses

All SPR experiments were performed at 25°C on a Biacore T100 instrument with the T200 sensitivity enhancement (GE Healthcare). For analyses of the binding of 4E10 and GEP Fv analytes to chip-captured ES ligands (**Fig. 2D** **,** **Table 1**), ESs at ∼1 µg/mL were captured on a SA sensor chip (GE Healthcare) following either carboxy (T117) or amine (T72, T93, T344) biotinylation, following the manufacturer's recommended protocol (EZ-Link, Thermo Scientific), in a running buffer of HBS-EP+ (10 mM HEPES, pH 7.4, 150 mM NaCl, 3 mM EDTA, 0.05% P-20; GE Healthcare) plus 0.1 mg/mL bovine serum albumin. A reference flow cell was left blank. Duplicate 4E10 and GEP Fv analyte injections were randomized and run at a flow rate of 50 µL/minute. Regeneration, if needed, was achieved by injection of 10 mM glycine at 50 µL/minute followed by buffer stabilization. Sensorgrams obtained from SPR measurements were double-reference subtracted [69] with BIAevaluation 2.0.3 software (GE Healthcare) employing previously described methodology [18]; data were fit with either 1∶1 or steady-state binding models. For analyses of the binding of 4E10 and GEP Fv analytes to gp140_3_ (**Fig. 2C**), gp140_3_ at 30 µg/mL in 10 mM sodium acetate (pH = 5.0) were direct amine coupled at a density of ∼2200 RUs on a CM5 sensor chip (GE Healthcare); a reference surface was prepared by activating and deactivating a flow cell without the addition of protein; gp140_3_ were prepared as previously described [18]. Duplicate 5 minute, 300 nM injections of 4E10 and GEP Fv were made in HBS-EP+ at 50 µL/minute followed by 5 minutes of dissociation, regeneration with 10 mM glycine (pH = 1.5) for 5 seconds and then 6 minutes of stabilization. Assays of Fab b12 or Fab 447-52D binding to SF162 gp140_3_ in the presence or absence of 4E10 Fv (**Fig. 13**) were conducted in HBS-EP+ buffer. Using standard amine coupling chemistry, 1273 RUs of SF162 gp140_3_ were coupled to a CM5 sensor chip. A reference surface was prepared by activating and deactivating a flow cell without the addition of protein. Two samples were injected at a flow rate of 50 µL/minute using the dual injection command in the T100 control software (v2.0.3, GE Healthcare) with injection 1 at 5 minutes, injection 2 at 2 minutes and a final dissociation time of 5 minutes. Fab-alone curves were generated by injecting HBS-EP+ followed by an injection of 10 nM Fab b12 or 447-52D and double referenced by subtracting a dual injection of HBS-EP+ followed by HBS-EP+. Fab with 4E10 Fv curves were generated by injecting 3 µM 4E10 Fv (∼90 RUs bound) followed by an injection of 10 nM Fab b12 or 447-52D in the presence of 3 µM 4E10 Fv and double referenced by subtracting a dual injection of 3 µM 4E10 Fv followed by 3 µM 4E10 Fv. Optimal regeneration was achieved by injection of 10 mM glycine (pH = 1.5) at a flow rate of 50 µL/minute for 5 seconds followed by a 6 minute buffer stabilization phase. Sensorgrams were corrected by the double-subtraction method [69] in Scrubber 2.0b software (BioLogic Software). **Fig. 13** shows the second sample of each set of injections with baselines zeroed just prior to the second injection.

### Crystallization and crystallography

Crystals of GEPs and 4E10 Fv were grown by the hanging-drop vapor diffusion method at 25°C with the following well solutions:

GEP 1: 25% w/v PEG 3350, 0.2 M NaCl, 0.1 M Tris (pH = 8.5)
GEP 1/T117: unbuffered 12% w/v PEG 8000, 0.1 M KCl, 5% v/v glycerol
GEP 2/T117: 1.6 M Li_2_SO_4_, 0.1 M sodium acetate (pH = 5.0)
GEP 7: 20% w/v PEG 2000, 0.2 M Trimethylamine N-oxide, 0.1 M Tris (pH = 9.0)
GEP 7/T117: 10% w/v PEG 10000, 0.1 M NH_4_OAc, 0.1 M Bis-Tris (pH = 5.5)
1C6: 3.5 M sodium formate, 0.1 M Bis-Tris Propane (pH = 7.0)

Crystals were cryopreserved in mother liquor containing 15% v/v glycerol (GEP 1, GEP 1/T117, GEP 7/T117), mother liquor containing 10% (1C6) or 20% (GEP 7) v/v glycerol, or 20% w/w sucrose (GEP 2/T117). Diffraction data for GEP 1, 4E10, and 1C6 were collected at the Advanced Light Source beamline 5.0.2 (Lawrence Berkeley National Laboratory, Berkeley, CA) and reduced using HKL-2000 [70] or d*TREK (Rigaku) [71]. Diffraction data for GEP 7, GEP 1/T117, GEP 7/T117 and GEP 2/T117 were collected in house with CuKα radiation on a R-AXIS IV++ image plate detector with HR optics (Rigaku) at −170°C. Initial phase information for all data sets was determined by molecular replacement, as implemented in the CCP4i program suite [72]–[74], using 3LF6.pdb (T117), 3LH2.pdb (GEPs) and 1JP5.pbd (1C6) as initial search models. Phases were improved by subsequent rounds of model building and refinement using COOT [75] and REFMAC [76]. Structure validation was carried out with PROCHECK [77], the MolProbity server [78], and the RCSB ADIT validation server. Data collection and structure refinement statistics are shown in **Table 2**. Crystals of GEP 2 alone could not be grown, despite considerable effort.

### PhIP-Seq analysis

4E10, GEP and 1C6 Fvs were coupled to beads and analyzed in duplicate. For each Fv analyzed, 3 mg of magnetic beads (Invitrogen M-270 Epoxy Dynabeads) were resuspended in 60 µL 0.1 M NaPO_4_ (pH = 7.4). Beads were rocked at ambient temperature for 24 hrs with 60 µg of each Fv in 1 M (NH_4_)_2_SO_4_ and then washed with 10 mM glycine in PBS to cap unreacted epoxy groups. Activity of Fv coupled beads was confirmed by epitope-scaffold binding prior to PhIP-Seq analyses. PhIP-Seq analyses were performed as previously described [17], [50]. Results were plotted as replicate #1 versus replicate #2 −Log_10_ *P*-values; highly discordant, and therefore spurious, hits falling near the axes were excluded from analysis. 241 peptides were also discarded because they displayed nonspecific binding to all Abs tested [17]. Peptide hydrophobicity was determined with the Sigma-Aldrich PEPscreen Library Design Tool and overall charge at neutral pH was determined with the Innovagen Peptide Property Calculator.

### Accession numbers

Coordinates and structure factor amplitudes have been deposited in the Protein Data Bank [79] under accession codes: 4M8Q.pdb (ligand-bound GEP 1), 4LRN.pdb (unbound GEP 1), 4M62.pdb (ligand-bound GEP 2), 4ODX.pdb (ligand-bound GEP 7), 4OB5.pdb (unbound GEP 7), and 4LCI.pdb (1C6).

## Supporting Information

Movie S1 Superposition of epitope binding site contacts from bound and unbound forms of 4E10. Residues from epitope binding site contacts are represented as mesh, pink for bound and yellow for unbound. The heavy chain CDR1 and CDR3 loops are shown in cartoon representations with residues W100H, W100bH, and F29H shown in licorice stick representations.
(M4V)

Movie S2 Superposition of epitope binding site contacts from bound and unbound forms of GEP 7. Residues from epitope binding site contacts are represented as mesh, pink for bound and yellow for unbound. The heavy chain CDR1 and CDR3 loops are shown in cartoon representations with residues W100H, W100bH, and F29H shown in licorice stick representations.
(M4V)
